# Workshop summaries from the 2024 voice AI symposium, presented by the Bridge2AI-voice consortium

**DOI:** 10.3389/fdgth.2024.1484818

**Published:** 2024-10-30

**Authors:** Ruth Bahr, James Anibal, Steven Bedrick, Jean-Christophe Bélisle-Pipon, Yael Bensoussan, Nate Blaylock, Joris Castermans, Keith Comito, David Dorr, Greg Hale, Christie Jackson, Andrea Krussel, Kimberly Kuman, Akash Raj Komarlu, Jordan Lerner-Ellis, Maria Powell, Vardit Ravitsky, Anaïs Rameau, Charlie Reavis, Alexandros Sigaras, Samantha Salvi Cruz, Jenny Vojtech, Megan Urbano, Stephanie Watts, Robin Zhao, Jamie Toghranegar, the Bridge2AI-Voice Consortium, Yael Bensoussan

**Affiliations:** ^1^ Department of Communication Sciences & Disorders, University of South Florida, Tampa, FL, United States; ^2^ Center for Interventional Oncology, Clinical Center, National Institutes of Health (NIH), Bethesda, MD, United States; ^3^ Computational Health Informatics Lab, Department of Engineering Science, Institute of Biomedical Engineering, University of Oxford, Oxford, United Kingdom; ^4^ Department of Medical Informatics & Clinical Epidemiology, Oregon Health & Science University, Portland, OR, United States; ^5^ Faculty of Health Sciences, Simon Fraser University, Burnaby, BC, Canada; ^6^ Department of Otolaryngology, University of South Florida, Tampa, FL, United States; ^7^ Canary Speech, Provo, UT, United States; ^8^ Whispp, Leiden, Netherlands; ^9^ Walt Disney Parks and Resorts, Orlando, FL, United States; ^10^ Office of Health Information and Data Science, Washington University in St. Louis, St. Louis, MO, United States; ^11^ Dysphonia International, Itasca, IL, United States; ^12^ Department of Laboratory Medicine & Pathobiology, University of Toronto, Toronto, Ontario, Canada; ^13^ Department of Otolaryngology–Head and Neck Surgery, Vanderbilt University Medical Center, Nashville, TN, United States; ^14^ The Hastings Center, Garrison, NY, United States; ^15^ Department of Otolaryngology, Weill Cornell Medicine, New York, NY, United States; ^16^ Department of Physiology and Biophysics, Weill Cornell Medicine, New York, NY, United States; ^17^ Department of Speech, Language, & Hearing Sciences, Boston University, Boston, MA, United States

**Keywords:** audiomics, voice biomarker, voice biomarkers, artificial intelligence, artificial intelligence—AI, ethical AI, Bridge2AI, Bridge2AI-Voice

## Abstract

**Introduction:**

The 2024 Voice AI Symposium, presented by the Bridge2AI-Voice Consortium, featured deep-dive educational workshops conducted by experts from diverse fields to explore the latest advancements in voice biomarkers and artificial intelligence (AI) applications in healthcare. Through five workshops, attendees learned about topics including international standardization of vocal biomarker data, real-world deployment of AI solutions, assistive technologies for voice disorders, best practices for voice data collection, and deep learning applications in voice analysis. These workshops aimed to foster collaboration between academia, industry, and healthcare to advance the development and implementation of voice-based AI tools.

**Methods:**

Each workshop featured a combination of lectures, case studies, and interactive discussions. Transcripts of audio recordings were generated using Whisper (Version 7.13.1) and summarized by ChatGPT (Version 4.0), then reviewed by the authors. The workshops covered various methodologies, from signal processing and machine learning operations (MLOps) to ethical concerns surrounding AI-powered voice data collection. Practical demonstrations of AI-driven tools for voice disorder management and technical discussions on implementing voice AI models in clinical and non-clinical settings provided attendees with hands-on experience.

**Results:**

Key outcomes included the discussion of international standards to unify stakeholders in vocal biomarker research, practical challenges in deploying AI solutions outside the laboratory, review of Bridge2AI-Voice data collection processes, and the potential of AI to empower individuals with voice disorders. Additionally, presenters shared innovations in ethical AI practices, scalable machine learning frameworks, and advanced data collection techniques using diverse voice datasets. The symposium highlighted the successful integration of AI in detecting and analyzing voice signals for various health applications, with significant advancements in standardization, privacy, and clinical validation processes.

**Discussion:**

The symposium underscored the importance of interdisciplinary collaboration to address the technical, ethical, and clinical challenges in the field of voice biomarkers. While AI models have shown promise in analyzing voice data, challenges such as data variability, security, and scalability remain. Future efforts must focus on refining data collection standards, advancing ethical AI practices, and ensuring diverse dataset inclusion to improve model robustness. By fostering collaboration among researchers, clinicians, and technologists, the symposium laid a foundation for future innovations in AI-driven voice analysis for healthcare diagnostics and treatment.

## Workshop 1: Speaking as One: International Standards Initiatives for Vocal Biomarker Data, sponsored by ASTM

**Objectives:** The educational workshop addressed concerns about duplication of effort and siloing of information that hinder the progress of audio-based biomarker technology. It showcased how international standardization initiatives could unite various stakeholders—from researchers and clinicians to entertainment companies and government agencies—to establish guidelines accelerating medical research, advancing revolutionary clinical trial technologies, and facilitating the promising future of decentralized science.

**Presenters:**

• Greg Hale: VP and Chief Safety Officer, Walt Disney Parks and Resorts
• Keith Comito, MS: President at Lifespan Extension Advocacy Foundation, Lead Software Engineer—Tech Lead, Advanced Research at Disney Streaming Services

**Presentation Summaries:**

**Greg Hale:** The session began with an overview of ASTM's role in international standards development and Disney's participation in those efforts. The discussion then transitioned into a detailed examination of how Disney has integrated advanced technologies to enhance guest experiences and safety. Hale shared his extensive experience in designing and engineering at Disney, including the development of accessibility technologies for hearing and visually impaired guests. He emphasized Disney's innovative use of captioning and audio descriptions synchronized with ride experiences to assist these guests.

**Keith Comito:** Keith Comito began his talk by addressing the significant impact COVID-19 had on Disney's global operations. The pandemic led to the shutdown of their cruise lines and parks worldwide, prompting him and Greg to work together on solutions. Comito, who was involved with Disney Streaming and ESPN analytics, collaborated with Hale to begin developing video and audio biomarker technology to help detect COVID and realized that the technology could be utilized well beyond COVID. Comito then described how one of the factors impeding the development of voice-based detectors for COVID was the lack of standardized data collection, and the consequent inability for shared data to be used by all parties to train effective machine-learning models.

Comito then highlighted the importance of collecting longitudinal data using various hardware, such as iPhones and webcams, and the creation of standardized protocols for data collection. This would enable multiple entities to collect and share data effectively. He emphasized the need for optional layers of data collection and standardization to accommodate differing collection scenarios and available hardware.

The discussion shifted to AI and data privacy, with Comito underscoring the role of healthcare institutions as data custodians and the challenges of retaining privacy in centralized systems. Emerging technologies like homomorphic encryption and zero-knowledge proofs were mentioned as potential solutions for secure data operations. Comito shared Disney's interest in sharing the capability to use biomarkers for health diagnostics in various settings. He described the potential of video and audio data in the creation of biomarkers able to identify individuals in distress, during events like marathons, where paramedics currently rely on visual assessment.

Hale then discussed the ongoing effort to harmonize global amusement standards, aligning ISO with EN and ASTM. He stressed the importance of expert collaboration across different standards organizations, noting that it has been a constant effort for over 20 years.

Comito then discussed additional health-related use cases for audio-based biomarkers, such as determining the difference between chronological age and biological age, the utility of such determinations in relation to health assessments, and the consequent intersection with the work of nonprofits such as the Lifespan Extension Advocacy Foundation (aka Lifespan.io).

Comito's talk provided a comprehensive overview of the challenges and solutions related to data collection and privacy, and the importance of ongoing collaboration in developing global standards.

## Workshop 2: Vocal Biomarker Solutions in the Wild, sponsored by Canary Speech

**Objectives:** The educational workshop emphasized that creating accurate vocal biomarker models was just the first step toward developing practical solutions for clinical and other settings. It reviewed various additional tasks and factors necessary for real-world implementation, including handling variations in speakers and acoustic environments, ensuring privacy and security, and employing machine learning operations (MLOps). Participants explored challenges in deploying vocal biomarker solutions, learned about measures beyond accuracy that should be considered, and discussed approaches for managing diverse audio data. They also gained insights into MLOps techniques for optimizing vocal biomarker solutions.

**Presenters:**

• **Nate Blaylock, PhD:** CTO of Canary Speech

**Presentation Summaries:**

**Nate Blaylock**: At the Voice AI Symposium, Dr. Blaylock presented a comprehensive overview of the challenges and techniques involved in moving from a vocal biometrics model to a full commercial solution. In particular, the presentation focused on considerations for processing voice data outside the lab, embedding the model in a full solution, and ensuring security and privacy. He also gave examples of approaches for many of these issues.

He emphasized the many ways that captured audio data in the field can differ from that collected in the lab, mentioning issues such as differences in recording hardware and software and varying acoustic environments. He suggested wider data collection as well as data augmentation techniques from the speech recognition field as possible approaches to this.

He then discussed the need to serve the model in a full solution, including the use of APIs as well as full applications. Additionally, he went over other needs such as traffic scalability, compute cost tracking, regional cloud deployments, and the use of service level indicators such as availability and latency. Throughout his presentation, Dr. Blaylock highlighted the practicalities of integrating various international standards and certifications, such as HITRUST and ISO 27001, into organizational practices. He noted the complexities of adhering to these standards, which often require rigorous audits and can introduce significant procedural friction. Moreover, he addressed the specific challenges of using cloud services and the need for continuous security measures to protect against threats and ensure compliance with health and privacy regulations, such as HIPAA and GDPR. He concluded with a call for partnerships and collaborations to advance the application of speech technology across different sectors, inviting attendees to a demo session showcasing their latest innovations in speech APIs and telehealth applications.

Dr. Blaylock's talk provided valuable insights into the realities of transforming vocal biomarker models into commercially viable solutions, underscoring the critical importance of handling audio variability, machine learning operations, and privacy and security in the development and deployment of these technologies.

## Workshop 3: Harnessing AI to Empower People with Voice Disorders, sponsored by Whispp and Dysphonia International

**Objectives:** The educational workshop, a collaboration between Whispp, an assistive technology company, and Dysphonia International, showcased how artificial intelligence (AI) has been enhancing the quality of life for affected individuals. It provided valuable insights into the intersection of AI and assistive technologies. The workshop featured a blend of lectures and group discussions that actively engaged participants. Attendees interacted with experts in the field, shared their perspectives, and explored innovative AI-driven solutions that are shaping the future of voice disorder management.

**Presenters:**

• **Megan Urbano, BM, MS, CCC-SLP**: Speech Pathologist USF Voice Center
• **Kimberly Kuman:** Executive Director of Dysphonia International
• **Akash Raj Komarlu:** Co-Founder and CTO of Whispp
• **Joris Castermans:** Co-Founder and CEO of Whispp
• **Charlie Reavis**: President of Dysphonia International

**Presentation Summaries:**

**Kimberly Kuman**: Kimberly Kuman provided an overview of the session and an introduction to Dysphonia International. The organization is dedicated to improving the lives of people with spasmodic dysphonia and related voice conditions through research, education, awareness, and support. She shared some of the challenges that people with voice disorders face and emphasized the importance of involving them in development of innovative products to help ensure that they can meet their specific needs to improve their quality of life.

**Megan Urbano**: Voice Specialized Speech Pathologist Megan Urbano discussed the differences between voice and speech. In short, voice was determined to be the sound signal as shaped by 3 subsystems—respiratory, phonatory, and resonatory. The respiratory system is comprised of the lungs and the flow of air through the vocal folds. High airflow produces a breathy quality, while low airflow can produce either a strained or rough vocal quality. The phonatory system is isolated to the vocal folds and the sound wave they create when they come together. Furthermore, vocal folds shape pitch (high and low) and vocal intensity (loud and quiet). Finally, the resonatory system shapes the remainder of the vocal tract to dampen or amplify soundwaves, creating a perception of the locus of a sound (e.g., a “throaty” vs. “nasal” sound). This was contrasted against speech, which can also be thought of as articulation, or how sound is shaped into language. An example given was as follows; Imagine a person speaking in English and then the same person speaking in Spanish. While their voice quality should be the same, the articulation, and therefore their speech, will be altered.

**Akash Raj Komarlu:** Akash summarized the current state of AI speech technology and how it can be used for people with voice disorders.

**Joris Castermans:** Joris Casterman shared his personal journey with stuttering and how that led to the development of a successful innovation in the assistive technology field with an app called Whispp. He shared his startup learnings and 6 key success factors which include: having a personal intrinsic motivation and drive, having a team with intrinsic motivation and drive, knowing and being close to your target group, never underestimating the importance of good UI design, having a unique and clear positioning of your solution and company, embracing the experts and your ambassadors. He concluded the presentation with a powerful video of people sharing the impact that voice disorders have on every aspect of their lives.

**Charlie Reavis:** Charlie Reavis concluded the workshop by expressing his gratitude to all the speakers for their dedication to helping people with voice conditions. As someone living with spasmodic dysphonia, he appreciated seeing the partnership between professionals and those affected by voice disorders.

## Workshop 4: Bridge2AI-Voice Best Practices

**Objectives:** The workshop, led by the Bridge2AI Voice Consortium, provided participants with an in-depth exploration of advanced methodologies and best practices essential for acquiring high-quality voice data in AI/ML model development for biomedicine. It covered the technical aspects of voice data acquisition, such as signal processing techniques, data annotation accuracy, and the integration of medical knowledge to ensure precise and relevant diagnostics and therapeutic applications. The session highlighted the importance of diverse datasets for improved model robustness and explored their application in clinical settings. Additionally, it emphasized ethical guidelines and the need for responsible AI use in healthcare. Participants learned about the technical foundations of voice data collection and how to determine clinical validation, while also gaining insights into Bridge2AI Voice best practices. They were equipped to identify these best practices, assess the quality of clinical validation used in tool development, and apply these principles to their own work.

**Presenters:**

• **Maria Powell, PhD, CCC-SLP:** Research Assistant Professor, Department of Otolaryngology- Head & Neck Surgery
• **Vardit Ravitsky, PhD:** President and CEO of The Hastings Center
• **Anaïs Rameau, MD MSc MS MPhil:** Assistant Professor, Department of Otolaryngology- Head & Neck Surgery, Weill Cornell Medicine
• **Yael Bensoussan, MD, MSc:** Assistant Professor, Department of Otolaryngology- Head & Neck Surgery, University of South Florida
• **Alexandros Sigaras, MS:** Assistant Professor of Research in Physiology and Biophysics, Weill Cornell Medical College
• **Jordan Lerner Ellis, PhD, FACMG, FCCMG:** Associate Professor, University of Toronto; Head and Director of the Advanced Molecular Diagnostics Laboratory, Mount Sinai Hospital

**Presentation Summaries:**

**Maria Powell:** Dr. Powell introduced herself and the other presenters as well as their roles in the program. She emphasized the importance of audience interaction and feedback, though the session had numerous presentations to cover.

**Vardit Ravitsky:** Dr. Ravitsky addressed the challenges of obtaining informed consent for AI-powered voice data collection. She highlighted the complexity of ensuring consent is truly informed given the unpredictable future uses and re-identification risks associated with the data. This uncertainty makes it difficult to fully inform participants about the potential implications of their participation. Dr. Ravitsky detailed the protocols for categorizing patient data into four diagnostic groups: mood disorders, respiratory disorders, voice disorders, and neurological disorders. Each group involves specific assessments and speech tasks. For mood disorders, the protocols include the PANAS, custom affect scale, DSM-5 criteria for PTSD and ADHD, and open-ended speech tasks. Respiratory disorders protocols involve different indices in the LCQ, breath sounds, and cough sounds. For voice disorders, the protocols include quality of life questionnaires, personal severity ratings, and specific speech tests. Neurological disorders protocols feature the Winograd task, word-color Stroop test, productive vocabulary tests, and the retelling of the Cinderella story.

Dr. Ravitsky emphasized the importance of clinical validation to ensure diagnostic accuracy based on current gold standards. She aims to confirm diagnoses through medical records while respecting privacy concerns. However, the lack of standardized diagnostic tests for some conditions poses challenges. Practical considerations include front-loading voice samples before medical procedures and adapting protocols in real-time. Dr. Ravitsky also stressed the need for sensitivity to emotional responses in open-ended speech tasks.

**Maria Powell:** Dr. Powell discussed the concept of hypothesis-agnostic protocols, which are central to their work. These protocols are designed to be flexible and applicable to multiple hypotheses. Currently, they have four protocols in play for mood, voice, respiratory, and neurological disorders. The goal is to create a protocol that can accommodate various hypotheses, achieved through their protocol development process. Dr. Powell explained that they aim to gather as much information as possible from medical records. While clear diagnoses can confirm the data, they may not always have the gold standard diagnostic tests. They document this gap and supplement it with other standards, such as MRI scans, to provide additional context.

In terms of patient recruitment, they focus on those who have the target diagnoses. For example, patients with breathy voices are included under voice disorders, even if they don't have a specific sound source. This approach ensures they capture relevant data across different conditions. The protocol includes a master list of phrases and questions, with specific subsets for each diagnostic group. All participants complete a core set of tasks, and additional tasks are based on their specific diagnoses. They have the capability to shuffle tasks but currently prioritize practical considerations, such as front-loading voice samples for patients who might be pulled into procedures. Dr. Powell acknowledged the practical challenges of collecting data in a clinical setting, particularly with patients undergoing treatments. They aim to improve this process in the future, ensuring a comprehensive and flexible approach to data collection across different disorders.

**Yael Bensoussan:** Dr. Bensoussan introduced the session by noting that the work being discussed was conducted by Ruth Bahr and Shaheen Awan and their team. She praised their expertise in acoustics, highlighting their extensive lab experience. The team had conducted experiments to compare different microphones, including iPhones, tablets, and high-end equipment, using a Kemar, a medical mannequin designed to simulate human vocal resonance. They tested various microphones and found that maintaining a consistent distance from the microphone was crucial for reliable data. Changes in distance significantly affected the accuracy of measurements, which was particularly noticeable with nasal interference and plosives when the microphone was too close to the mouth. Their recommendation for clinical studies was to use headset microphones to ensure a consistent distance. They found that a distance of about three inches from the mouth provided the best sound quality without interference. They also emphasized the importance of using gain adjustment platforms and integrating technology to automatically adjust microphone gain based on distance and volume.

**Alexandros Sigaras:** Alexandros Sigaras began by acknowledging that he had the privilege of leading the team responsible for building tools to ensure participants’ voices are heard. One of his key responsibilities is addressing ethical concerns. He asked the audience how many of them build tools, engaging those involved in similar work. Sigaras discussed the importance of feasibility, accessibility, and scalability in tool development. He shared that a significant challenge was ensuring participants could correctly identify and press the recording button, as about 80% initially struggled with this. He emphasized the value of feedback from clinicians and participants, which has been crucial in improving the tools.

He highlighted their use of the Kemar mannequin for acoustic testing, which helps ensure reproducibility. They tested various devices, noting that microphone gain differs across devices like iPads and iPhones. Sigaras mentioned the differences in the number of microphones across Apple devices, which affects audio quality. The team measured headset performance to prevent audio clipping and found that maintaining the right distance is key for accurate data collection. This approach benefits both remote and clinical data collection settings.

Sigaras concluded by discussing scalability, particularly within the Apple ecosystem. Sigaras emphasized the goal of making the tools accessible to all participants, whether or not they have a voice disorder. Remote enrollment and consent are already live, with ongoing efforts to improve remote data collection. He invited participants to enroll and contribute to the Bridged AI program.

**Jordan Lerner-Ellis:** Dr. Lerner-Ellis focused on the challenges and strategies for recruiting and enrolling patient participants. He outlined the criteria for establishing high-volume expert clinics, which are specialized units at participating institutions with high patient volumes for specific diseases. Despite these clinics’ potential, success can vary due to factors like remote clinic locations.

Dr. Lerner-Ellis shared experiences from sites like Bridgepoint and Baycrest in Toronto, noting that even high-volume clinics like those at the Center for Addition and Mental Health, sometimes recruit only one or two new participants per month. Recruiting from some jurisdictions such as In Ontario, where there are over 140 hospitals, benefits from centralized research ethics board approvals through systems like clinical trials Ontario (CTO), which is being taken advantage of. He emphasized the importance of specialized focus and partnerships, such as with Life Labs for remote and collection of blood samples. He mentioned that recruiting Alzheimer's disease patients often requires substitute decision-makers and is more effective in the inpatient settings. Dr. Lerner-Ellis pointed out the need for additional staff to assist with coordination and data collection, as clinicians are often too busy to handle these tasks themselves. Dr. Lerner-Ellis emphasized the importance of providing research assistants to facilitate data collection and ensuring the involvement of necessary staff for successful recruitment and data gathering. In conclusion, he invited attendees to the tech fair and poster session for further engagement and networking.

## Workshop 5: Unlocking the Secrets of Voice: A Deep Dive into Physiology and Signal Processing

**Objectives:** The educational workshop provided a comprehensive understanding of voice analysis by exploring three key domains. First, it distinguished between voice and speech, examined how the ear perceived sound vs. what acoustic data revealed, and assessed quality ratings to demonstrate how AI could enhance voice analysis. Second, it delved into available acoustic measures, their significance, multi-dimensionality, and the pros and cons of existing methods, illustrating the need for AI-driven approaches. Lastly, it showcased how deep learning and transformer models encoded nuanced variability in speech, emphasizing how to manage potential information loss in spectrograms and interpret AI models trained on voice, while offering strategies to make Voice AI more explainable.

**Presenters:**

• **Ruth Bahr, PhD, CCC-SLP, BCS-CL:** Professor, Communication Sciences and Disorders, University of South Florida
• **Jenny Vojtech, PhD:** Research Assistant Professor, Department of Speech, Language, & Hearing Sciences Boston University
• **James Anibal:** DPhil student at Reuben College, an NIH-Oxford Scholar, and a bioinformatics instructor at the Foundation for Advanced Education in the Sciences

**Presentation Summaries:**

**Ruth Bahr:** Dr. Bahr began her presentation by discussing the profound nature of the human voice and the importance it holds in one's identity. She quoted Daniel Day-Lewis, who called the voice “the fingerprint of the soul,” emphasizing how personal and irreplaceable it is for individuals. She made a clear distinction between voice and speech, explaining that the voice is the sound produced by airflow and vocal fold movement, while the vocal tract modifies this sound to form speech. She explained the anatomy and physiology of the larynx, focusing on how the vocal folds vibrate to produce sound. She described the relationship between air pressure, airflow, and vocal fold movement in generating different acoustic signals. Dr. Bahr explored vocal biometrics, noting the unique variability of individual voices and the complexity involved in distinguishing pathological changes from intentional modifications. She concluded by underscoring the challenges of identifying biomarkers, given the variations in speech patterns and the emotional connection people have with their own voices.

**Jenny Vojtech:** Dr. Vojtech took the stage next, focusing on the role of acoustic measures in clinical voice assessment. She detailed the recommendations made by the American Speech-Language-Hearing Association (ASHA) for key acoustic measures like Sound Pressure Level (SPL), fundamental frequency, and cepstral peak prominence (CPP). She critiqued the continued use of measures like jitter and shimmer, which, despite being traditionally used, are no longer recommended due to their lack of clear physiological ties and poor handling of severe dysphonia. She highlighted the limitations of these acoustic measures, noting how environmental factors can impact recordings and how fundamental frequency and SPL are not always directly linked to what the ear perceives. She stressed the need for automating and validating these measures in clinical settings to ensure consistency and reliability. The adoption of these measures would provide a comprehensive supplement to perceptual assessments, enabling more accurate tracking of voice quality changes over time.

**James Anibal:** James Anibal followed, turning the conversation toward deep learning and artificial intelligence (AI) in voice analysis. He advocated for the power of AI to analyze the complex, nonlinear combinations of features in the human voice and correlate them with disease status or diagnostic methods. Anibal described the role of self-supervised learning in teaching AI models to detect patterns by predicting missing data points. He expanded on contrastive learning, an approach that identifies “same” and “different” pairs to refine model predictions, allowing the AI to focus on the nuances of voice biomarkers. Anibal acknowledged the challenges and opportunities in this domain, noting that while these models are powerful, they require vast amounts of data and face issues related to data privacy and the explainability of their outputs. Despite these challenges, he emphasized the potential of integrating multimodal models with existing language models like GPT to facilitate more nuanced and comprehensive voice analysis, especially for clinical purposes.

## The Bridge2AI-Voice consortium

Yael Bensoussan, University of South Florida, Tampa, FL, US; Olivier Elemento, Weill Cornell Medicine, New York, NY, USA; Anaïs Rameau, Weill Cornell Medicine, New York, NY, USA; Alexandros Sigaras, Weill Cornell Medicine, New York, NY, USA; Satrajit Ghosh, Massachusetts Institute of Technology, Boston, MA, USA; Maria Powell, Vanderbilt University Medical Center, Nashville, TN, USA; Vardit Ravitsky, University of Montreal, Montreal, Quebec, Canada; Jean Christophe Belisle-Pipon, Simon Fraser University, Burnaby, BC, Canada; David Dorr, Oregon Health & Science University, Portland, OR, USA; Phillip Payne, Washington University in St. Louis, St. Louis, MO, USA; Alistair Johnson, University of Toronto, Toronto, Ontario, Canada; Ruth Bahr, University of South Florida, Tampa, FL, USA; Donald Bolser, University of Florida, Gainesville, FL, USA; Frank Rudzicz, Dalhousie University, Toronto, ON, Canada; Jordan Lerner Ellis, University of Toronto, Toronto, ON, Canada; Kathy Jenkins, Boston Children's Hospital, Boston, MA, USA; Shaheen Awan, University of Central Florida, Orlando, FL, USA; Micah Boyer, University of South Florida, Tampa, FL, USA; Bill Hersh, Oregon Health & Science University, Portland, OR, USA; Andrea Krussel, Washington University in St. Louis, St. Louis, MO, USA; Steven Bedrick, Oregon Health & Science University, Portland, OR, USA; Toufeeq Ahmed Syed, UT Health, Houston, TX, USA; Jamie Toghranegar, University of South Florida, Tampa, FL, USA; James Anibal, University of South Florida, Tampa, FL, USA; Duncan Sutherland, New York, NY, USA; Enrique Diaz-Ocampo, University of South Florida, Tampa, FL, USA; Elizabeth Silberhoz, University of South Florida, Tampa, FL, USA; John Costello, Boston Children's Hospital, Boston, MA, USA; Alexander Gelbard, Vanderbilt University Medical Center, Nashville, TN, USA; Kimberly Vinson, Vanderbilt University Medical Center, Nashville, TN, USA; Tempestt Neal, University of South Florida, Tampa, FL, USA; Lochana Jayachandran, Mt. Sinai Health, Toronto, ON, Canada; Evan Ng, The Hospital for Sick Children, Toronto, ON, Canada; Selina Casalino, Mt. Sinai Health, Toronto, ON, Canada; Yassmeen Abdel-Aty, University of South Florida, Tampa, FL, USA; Karim Hanna, University of South Florida, Tampa, FL, USA; Theresa Zesiewicz, University of South Florida, Tampa, FL, USA; Elijah Moothedan, Florida Atlantic University, Boca Raton, FL, USA; Emily Evangelista, University of South Florida, Tampa, FL, USA; Samantha Salvi Cruz, Vanderbilt University Medical Center, Nashville, TN, USA; Robin Zhao, Weill Cornell Medicine, New York, NY, USA; Mohamed Ebraheem, University of South Florida, Tampa, FL, USA; Karlee Newberry, University of South Florida, Tampa, FL, USA; Iris De Santiago, University of South Florida, Tampa, FL, USA; Ellie Eiseman, University of South Florida, Tampa, FL, USA; JM Rahman, University of South Florida, Tampa, FL, USA; Stacy Jo, Boston Children's Hospital, Boston, MA, USA; Anna Goldenberg, Hospital for Sick Children, Toronto, ON, Canada

